# Supplementary material for: Human genetic variation reveals FCRL3 is a lymphocyte receptor for Yersinia pestis
Source: bioRxiv. 2024 Dec 8:2024.12.05.626452. Preprint. [Version 1] doi: 10.1101/2024.12.05.626452 (PMC11643160; doi:10.1101/2024.12.05.626452)
Supplement: Supplement 1 [file media-1.pdf]

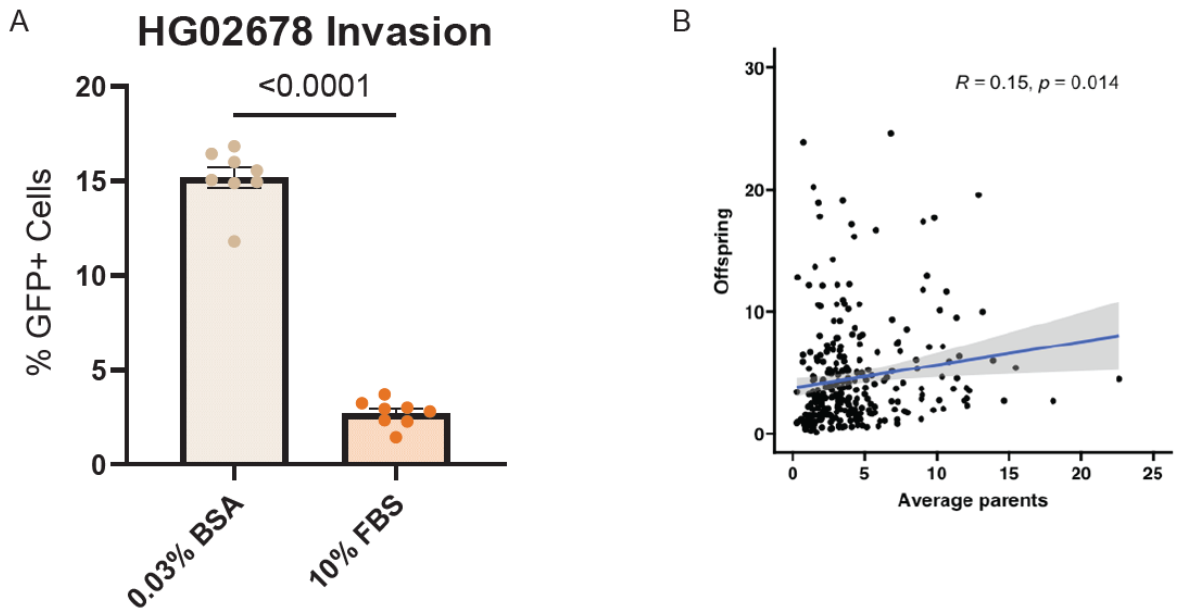

Figure S1. *Y. pestis* invasion into LCLs is inhibited by FBS and heritable. (**A**) Flow cytometric measurement of *Y. pestis* (KIM6+ +pMMB67GFP) invasion of LCLs at 4 hpi in RPMI media with either 10% FBS or 0.03% BSA after gentamicin protection assay. 2 experiments are grand mean normalized with 8 total replicates plotted for each condition. An unpaired t-test was performed to determine significance. (**B**) Parent-offspring linear regression of *Y. pestis* invasion into LCLs gives an estimated heritability of 0.15, a slope of 0.19 and a p-value = 0.014.

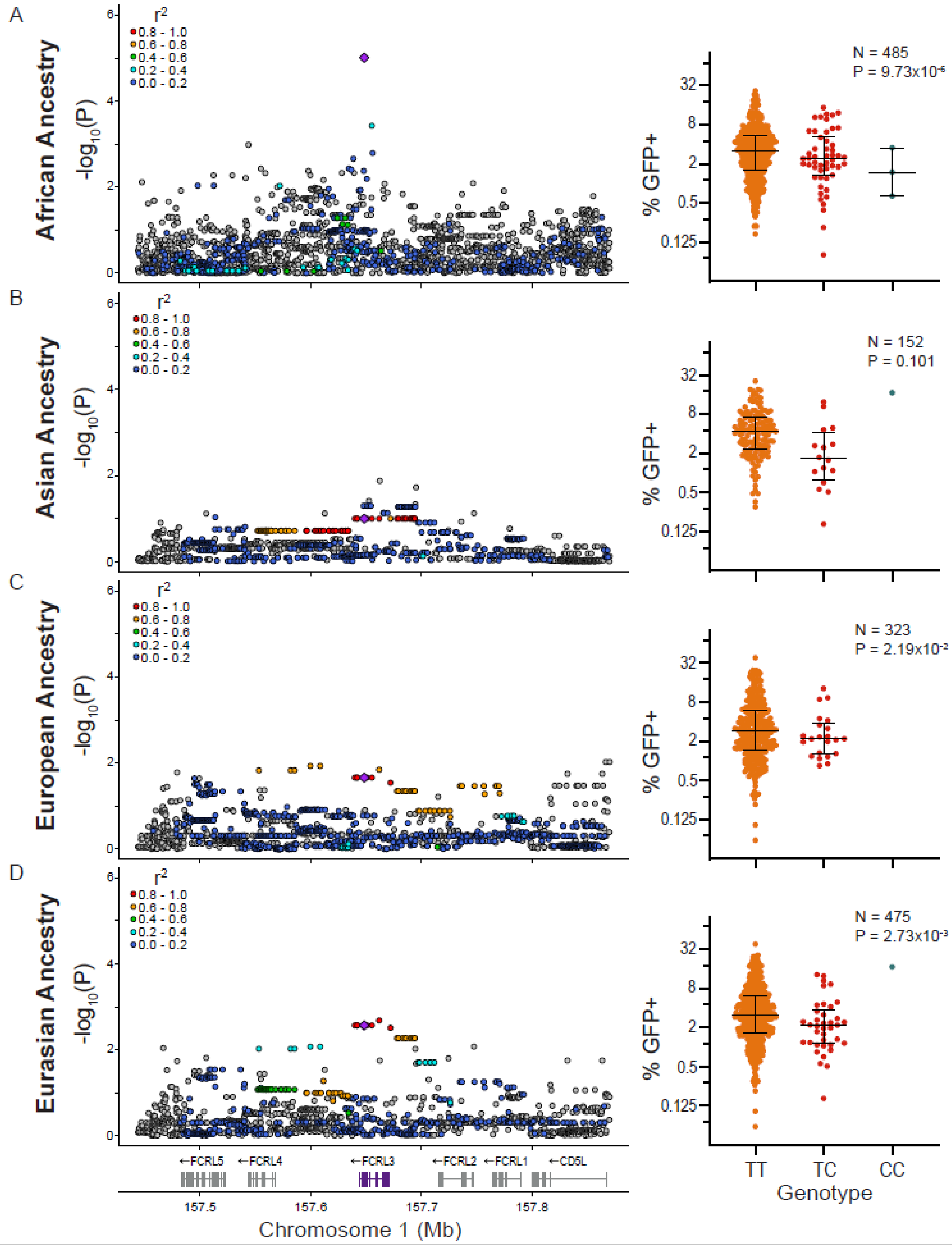
A
B
C
D
African Ancestry
Asian Ancestry
European Ancestry
Eurasian Ancestry
-log10(P)
r2
0.8 - 1.0
0.6 - 0.8
0.4 - 0.6
0.2 - 0.4
0.0 - 0.2
N = 485
P = 9.73x10-6
N = 152
P = 0.101
N = 323
P = 2.19x10-2
N = 475
P = 2.73x10-3
% GFP+
←FCRL5
←FCRL4
←FCRL3
←FCRL2
←FCRL1
←CD5L
157.5
157.6
157.7
157.8
Chromosome 1 (Mb)
TT
TC
CC
Genotype

Figure S2. Locus zoom and genotypic mean plots of LCLs stratified by ancestry. Local Manhattan plots and genotypic mean plots stratified by African (GWD, YRI, ESN), Asian (JPT, CHB, KHV), European (CEU, IBS), or EurAsian (JPT, CHB, KHV, CEU, IBS) ancestries. A purple diamond denotes rs2282284 and LD with SNPs in the locus is shown by red >= 0.8, orange = 0.6-0.8, green = 0.4-0.6, light blue = 0.2-0.4, dark blue < 0.2, and grey has no LD data. LD was determined by all populations within the stratified group. -$\log_{10}(p)$ values at the rs2282284 locus was plotted and phenotypes were plotted by genotype. All populations demonstrate the C allele of rs2282284 is associated with decreased invasion.

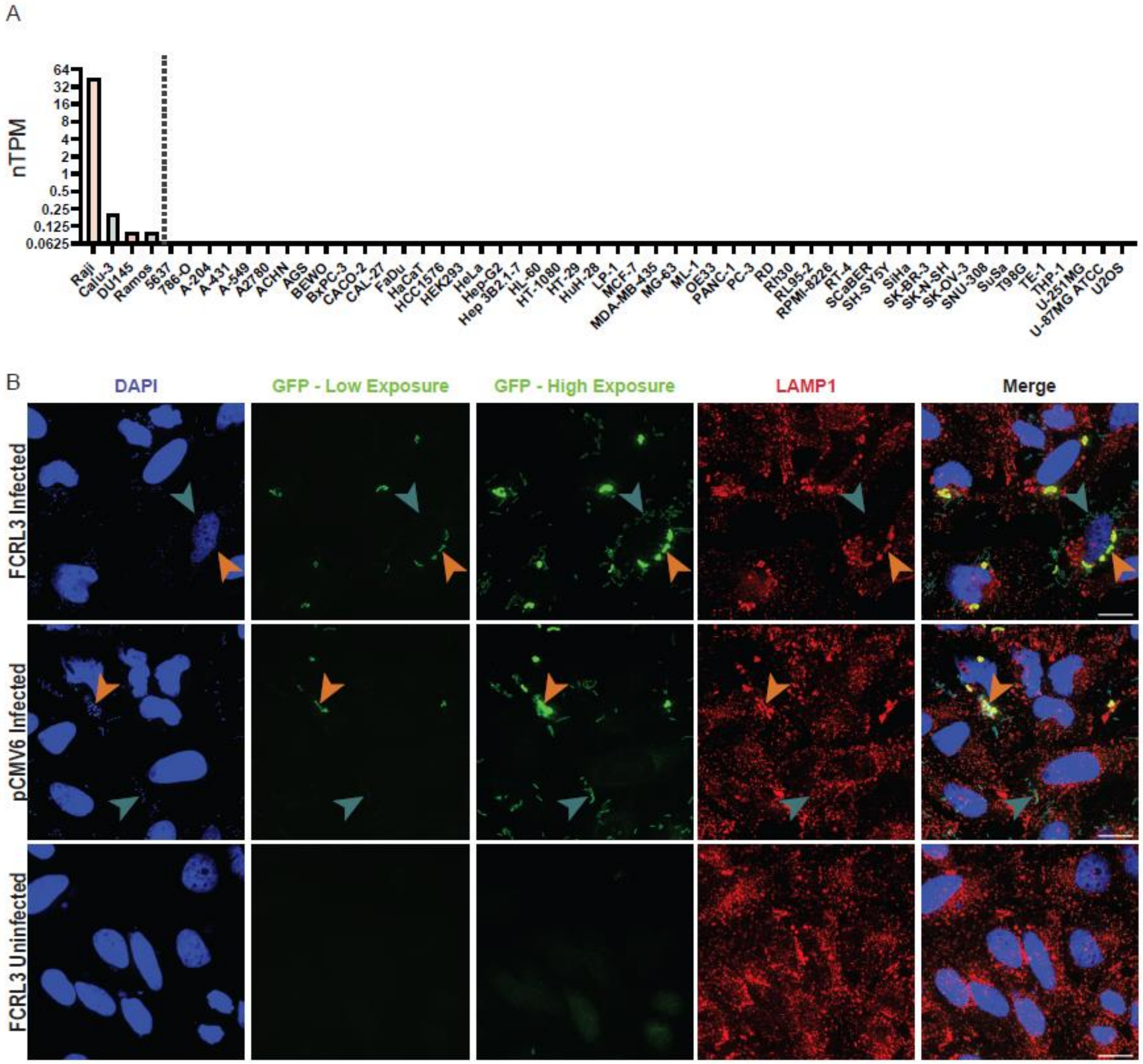

Figure S3. No FCRL3 protein is detected in HeLa cells and intracellular *Y. pestis* is in LAMP+ vesicles at 4 hpi. (**A**) Bar graphs of expression in common cell lines from the Human Protein Atlas (Uhlen et al., 2015) show expression only in lymphoma, Calu-3, and DU145 cell lines and no expression in HeLa. (**B**) GFP^{high} cells have intracellular *Y. pestis* within LAMP1+ vacuoles. HeLa cells transfected with empty vector or *FCRL3* plasmid were infected with KIM6+ +pMMB67GFP *Y. pestis* for 1 hr, treated with gentamicin for 1 hr, and induced with IPTG for 2 hr prior to fixation with 4% paraformaldehyde. After

incubating for 30 minutes in block/perm, DNA was stained with 2.5µM DAPI, LAMP1 was stained red with a 1:20 dilution of H4A3-s mouse LAMP1 antibody (Developmental Studies Hybridoma Bank), and bacteria are shown in green at high exposure to include the GFP^{low} population. GFP^{low} bacteria are indicated with a teal arrow head and GFP^{high} bacteria with an orange arrow head. Images were taken on a Zeiss Observer Z1 inverted microscope with a 63x water objective. A 20µm scale bar is located on the merged image.

Table S1. (separate file) *Y. pestis* invasion measurement for 961 LCLs (3 replicates and mean)

Table S2. sgRNAs and primers for CRISPR mutagenesis and validation

**GENE**	**GUIDE 1**	**GUIDE 2**	**GUIDE 3**	**FWD primer**	**REV primer**
**CD46**	GAGAAACAUGUCCAUAUAUA	AACUCGUAAGUCCCAUUUGC	UUGCUCCUUAGAGGAAAUAA	TGCCTGGGTGAATATGAATCTT	TGTCAGAAACAGCAAGTAGTTTTG
**FCRL3**	AAUUUCCAGGCUCUGUAAUU	GAUACCAAUAUGUGUCUCCC	CUGUGGACCAUGGAGGAUUG	TCTGCCTAGGATCCCTGCAT	ACCCTGGTCCTGACTGGA

Table S3. Plasmids

Plasmid backbone	insert	tag	Origin
pCMV6		MYC/FLAG	Simon Gregory
pCMV6	FCRL3	MYC/FLAG	Origene
pCMV-SPORT6	CD31		Tim Wilson
pFLAG-CMV-4	FCRL1	FLAG	Tim Wilson
pFLAG-CMV-6	FCRL3	FLAG	Tim Wilson
pFLAG-CMV-7	FCRL4	FLAG	Tim Wilson
pFLAG-CMV-8	FCRL5	FLAG	Tim Wilson
pFLAG-CMV-9	FCRL6	FLAG	Tim Wilson
pD649-Hasp-COMP5AP	CD31	AviTag/9xHis	Addgene
pD649-Hasp-COMP5AP	FCRL5	AviTag/9xHis	Addgene

Table S4. Oligonucleotides for Quikchange mutagenesis

mutation	Forward	reverse
N721S	catgaagaagatgatgaagaaagctatgagaatgtaccacgtgta	tacacgtggtacattctcatagctttcttcatcatcttcttcatg
Y650F	ggagctggagccaatgttcagcaatgtaaatcctg	caggatttacattgctgaacattggctccagctcc
Y662F	ctggagatagcaacccgattttttcccagatctg	cagatctgggaaaaaatcgggttgctatctccag
Y692F	gaggaacttacagtcctcttttcagaactgaagaagaca	tgtcttcttcagttctgaaaagaggactgtaagttcctc
Y722F	cccatgaagaagatgatgaagaaaactttgagaatgtaccac	gtggtacattctcaaagttttcttcatcatcttcttcatggg
